# Metals and Disease

**DOI:** 10.1155/2012/825354

**Published:** 2012-03-21

**Authors:** Ana-Maria Florea, Dietrich Büsselberg, David Carpenter

**Affiliations:** ^1^Institute of Neuropathology, Heinrich-Heine University Düsseldorf, 40225 Düsseldorf, Germany; ^2^Department of Physiology and Biophysics, Weill Cornell Medical College in Qatar, Qatar Foundation-Education City, P.O. Box 24144, Doha, Qatar; ^3^Institute for Health and the Environment, University at Albany, Rensselaer, NY 12144, USA

Metals are a part of our daily life. They are used for construction and for countless products (e.g., cars, mobile phones, or computers). Without the use of metals, human society would have not been developed to its current stage. While the raw materials are provided by our planet, humans learned to extract metals from mineral ore and to refine them.

As a consequence to their wide use in the modern world, we are all exposed to metals and metal compounds to a degree that was rare before the industrial age. Exposing the cell(s) of living organisms to elements which were hardly available during genesis of life causes multiple effects on those cells and ultimately human health will be affected.

Some metals serve critical functions in the human body. Iron is a necessary component of hemoglobin in red blood cells. Copper, manganese, zinc, cobalt, chromium, molybdenum, and selenium are all required as enzyme cofactors or prosthetic groups, and human disease results if the diet is deficient in the metals. However, exposure of humans to excessive levels of “physiologic” metals are associated with disease. Other metals, such as lead, aluminum, and arsenic, have no known beneficial effects in the human body and have only toxic actions. (This issue does not describe the positive health effects of some essential metals which are crucial to maintain normal body function.) 

This special issue presents the latest scientific insights into how metals influence and change cell and body function. One of the contributions of this special issue gives “a global primary health care perspective” linking the risks of metal exposure of humans to the contamination of the environment. The authors highlight the concern that primary care workers possibly underestimate occupational and environmental exposures to chemicals in clinical evaluations. The paper summarizes worldwide studies which explore the relationship between metal exposures and adverse health effects. Finally it suggests some guidelines to evaluate basic occupational and environmental exposure. 

Other two papers emphasize health effects of metals on the cardiovascular and the nervous system. Clearly both systems have a major impact on human health. The papers demonstrate that the functions of both systems are clearly impaired by metals. The paper on the cardiovascular system proposes a mechanism of action for interactions between genetic, nutritional, and environmental factors, while the paper on the nervous system emphasizes the multiple sites of action of a single metal at the pre- and postsynaptic terminal as well as the targets for effects which impair synaptic transmission and, therefore, learning and memory function. Not only does a single metal have many sites of action (and the most sensitive has to be defined), but also different metals might both share some targets and act at different targets. Therefore, the environmental exposure might result in additive effects. The authors conclude that the multiple effects of metals may occur simultaneously and are dependent on the specific metal species, concentrations, and the cell type involved.

Another paper in this special issue is somehow unique in this volume since it not only describes the negative health effects of metals but also illustrates that under certain specific circumstances metals might have beneficial effects and could be used to fight specific diseases (e.g., cancers). The authors conclude that metals could actually be both risk factors as well as healing agents for specific forms of breast cancer.

The diversity of molecular mechanisms modulated by exposure to arsenic and its relation to acute- and chronic-toxicity as well as in regard to cancer development is discussed in one of the papers of this special issue.

Two papers of this issue are research investigations dealing with exposure to lead or aluminum, respectively. The investigation on the early effects of long-term exposure to lead shows that mainly motor performance parameters are an early neurotoxic indicator of lead toxicity while the subjects exhibited a slowed poststrain resetting behavior of the vegetative nervous system, which correlated with the individual blood lead level.

The exposure to low concentrations of aluminum chloride on thymocytes and lymphocytes results in a rapid and dose-dependent injury of these cells illustrating that aluminum has cytotoxic effects on cells of the immune system.

Finally, there is a paper that takes a different point of view and gives some important general information for physicians on how they could recognize and prevent overexposure to methylmercury from fish and seafood consumption.

Overall, every single person gets in contact with metals in daily life. Some of these metals have considerable health effects. While most of these effects have negative consequences resulting in a large variety of different symptoms (which could be minor or severe); some of the biological properties of these metals and their compounds could be used to treat life-threatening diseases (e.g., cancer).

To understand the negative and positive effects of metals and metal compounds on human health and disease, more research is needed. Additionally the public should be educated and sensitized in order to minimize the environmental contamination and prevent metal-induced intoxications.

*Ana-Maria Florea* *Ana-Maria Florea* *Dietrich Büsselberg* *Dietrich Büsselberg* *David Carpenter* *David Carpenter*

